# Driving Down Mortality: A 12-Year Retrospective Cohort Analysis of Mechanical Power and Driving Pressure in Ventilated ICU Patients

**DOI:** 10.3390/medicina61091668

**Published:** 2025-09-14

**Authors:** Payam Rahimi, Sinan Aşar, Nuri Burkay Soylu, Tuğba Yücel Yenice, Emral Canan, Zafer Çukurova

**Affiliations:** 1 Department of Anesthesiology and Intensive Care, Bakirkoy Dr. Sadi Konuk Training and Research Hospital, University of Health Sciences, 34147 Istanbul, Turkey; tugbayucel09@gmail.com (T.Y.Y.); emralc@gmail.com (E.C.); zcukurova@gmail.com (Z.Ç.); 2 Department of Anesthesiology and Intensive Care, Mardin Training and Research Hospital, University of Health Sciences, 47100 Mardin, Turkey; sinanasaras@gmail.com; 3 Canakkale State Hospital, 17100 Canakkale, Turkey; drburkaysoylu@gmail.com

**Keywords:** driving pressure, mechanical power, ICU mortality, mechanical ventilation, lung and diaphragm protective ventilation

## Abstract

*Background and Objectives*: Mechanical ventilation, while essential, can precipitate ventilator-induced lung injury (VILI) due to excessive mechanical stress. Among respiratory mechanics, driving pressure (ΔP) has emerged as the most robust predictor of mortality, with mechanical power (MP) and tidal volume (TV), respiratory rate (RR), positive end-expiratory pressure (PEEP), and peak inspiratory pressure (P_peak_) also potentially influencing clinical outcomes. This study primarily evaluated whether the implementation of a standardized Lung and Diaphragm Protective Ventilation (LDPV) protocol, designed to minimize ΔP, reduced intensive care unit (ICU) mortality. Secondary objectives included assessing the prognostic impact of MP, P_peak_, TV, RR, and PEEP on mortality in the pre- and post-LDPV implementation periods. *Materials and Methods*: In this retrospective cohort study, a total of 3468 adult ICU patients receiving invasive mechanical ventilation between 2012 and 2024 were analyzed. Patients were categorized into two groups: pre-LDPV (2012–2018) and post-LDPV (2019–2024). Ventilatory data were automatically collected using the Metavision system and evaluated through receiver operating characteristic (ROC) derived cutoffs, survival modeling, and Cox proportional hazards regression. *Results*: Implementation of the LDPV protocol was associated with a significant reduction in ICU mortality (47.7% vs. 41.1%, *p* < 0.0001) and a shorter ICU length of stay. Patients in the post-LDPV cohort (2019–2024) exhibited lower ΔP (median 12.9 vs. 14.3 cmH_2_O), lower MP (median 15.0 vs. 17.0 J/min), improved respiratory system compliance, and reduced peak inspiratory pressure (P_peak_) and tidal volume (TV_e_) compared to the pre-LDPV cohort (2012–2018). Analysis revealed that the reduction in ΔP was the most significant determinant of improved survival; median ΔP decreased by approximately 2 cmH_2_O (from 14.3 to 12.9 cmH_2_O). Elevated MP and P_peak_ were also predictive of mortality, while compliance below 34 mL/cmH_2_O consistently indicated a poor prognosis across both study periods. *Conclusions*: Implementation of an LDPV protocol significantly reduced ICU mortality, primarily through the systematic reduction in ΔP, while MP and its components provided complementary prognostic information. These findings underscore ΔP as the primary modifiable determinant of survival, with MP, P_peak_, TV, and PEEP serving as secondary indicators of VILI.

## 1. Introduction

Mechanical ventilation, while critical for providing respiratory support in patients with Acute Respiratory Distress Syndrome (ARDS), paradoxically poses a risk of exacerbating lung injury through the pathogenesis of Ventilator-Induced Lung Injury (VILI). The dual mechanisms of volutrauma and barotrauma induce alveolar damage, which may trigger systemic inflammation and hinder recovery, particularly in pre-injured lungs [1]. Lung and Diaphragm Protective Ventilation (LDPV) has been shown to significantly improve survival [2]. However, beyond conventional parameters like tidal volume and plateau pressure, newer physiologic metrics have emerged to quantify the mechanical “stress” imposed by ventilation [3]. Among these, driving pressure (ΔP) has gained recognition as a critical determinant of outcome [4,5]. Amato et al. found ΔP to be the ventilatory variable most strongly associated with survival in ARDS, with each reduction in ΔP translating to a substantial mortality benefit [6].

More recently, mechanical power (MP), defined as the rate of energy transfer from the ventilator to the lung, has been proposed as a comprehensive index for assessing the risk of VILI [3]. Elevated MP reflects the combined injurious effects of high tidal volumes, airway pressures, and respiratory rates. MP is associated with increased mortality among mechanically ventilated intensive care unit (ICU) patients [7].

These findings indicate that reducing ΔP may represent the most effective strategy for improving survival in critically ill patients, while MP and other respiratory mechanics like peak inspiratory pressure (P_peak_), respiratory rate (RR), tidal volume (TV), and positive end-expiratory pressure (PEEP) provide supplementary prognostic information. Accordingly, the primary objective of this study was to evaluate whether ICU mortality decreased following the implementation of a standardized LDPV protocol focused on ΔP reduction. Secondary objectives included assessing the impact of MP and other respiratory mechanics on mortality during the pre- and post-LDPV periods and determining their relative prognostic contributions in conjunction with respiratory system compliance.

## 2. Materials and Methods

This study was approved by the University of Health Sciences, Bakırköy Dr. Sadi Konuk Training and Research Hospital Clinical Research Ethics Committee with protocol number 2025/24 and decision number 2025-02-17.

### 2.1. Study Design and Population

This retrospective, cohort study was conducted in Bakirkoy Dr. Sadi Konuk Training and Research Hospital, 30 beds general ICU between January 2012 and December 2024. We retrieved this dataset by utilizing structured query language queries (SQL) from the IMD Soft-Metavision/QlinICU Clinical Decision Support (Tel Aviv, Israel) system. All patients aged ≥18 years who received invasive mechanical ventilation for at least 24 h were included in the study. We excluded: 1. Patients with missing respiratory mechanics or ICU mortality data, 2. Those who received invasive mechanical ventilation for less than 24 h, 3. Patients who received non-invasive mechanical ventilation, 4. Patients with Extracorporeal support (ECMO or ECCO_2_R) treatments, 5. Patients with terminal illness (life expectancy < 48 h), 6. Pediatric patients, 7. Patients with COVID-19 diagnosis. Due to the exceptional and atypical clinical characteristics of patients admitted during the COVID-19 pandemic (2020–2022), which introduced significant heterogeneity in disease severity, comorbidities, and ventilatory management compared to non-COVID ICU populations, these patients were excluded from the final analysis. This exclusion was implemented to maintain the homogeneity of the study cohort and to ensure that temporal comparisons of mechanical ventilation strategies (pre- vs. post-LDPV implementation) were not confounded by the distinct pathophysiology and treatment protocols associated with COVID-19-related ARDS. In our ICU, the routine controlled ventilation strategy employed is pressure control ventilation (PCV) mode. Volume control ventilation (VCV) was utilized only under specific circumstances, including in patients with severe metabolic acidosis necessitating precise control of minute ventilation, or in cases requiring accurate tidal volume delivery (e.g., patients with status epilepticus or elevated intracranial pressure). All patients were mechanically ventilated using Maquet Servo-i,u devices (Getinge, Gothenburg, Sweden).

**Figure 1:**
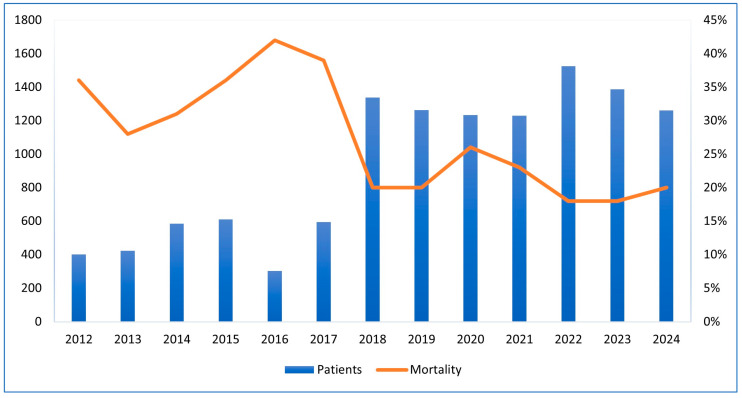
Trends in ICU Admissions and Mortality Rates (2012–2024).

The graph shows that ICU admissions rose from around 400 in 2012 to over 1500 in 2022. A sharp increase occurred in 2018, jumping from 600 to approximately 1350. Meanwhile, the ICU mortality rate decreased from 37% in 2012 to about 20% in 2018 and stayed stable through 2024. The highest mortality rate was over 40% in 2016, when admissions were around 600. This inverse trend after 2018 suggests improved healthcare outcomes despite rising admissions (Figure 1).

### 2.2. Study Periods

Following the publication of several studies in 2015–2016 demonstrating the association between reduced ΔP and MP and improved survival, LDPV protocol was established in our clinic. As of November–December 2018, this protocol has been implemented in all intubated patients.

To evaluate the impact of LDPV, we divided patients into two groups based on the implementation period of the LDPV strategy: The pre-LDPV group, from January 2012 to December 2018, and the Post-LDPV group, from January 2019 to December 2024. The LDPV protocol, introduced at the end of 2018 for all intubated patients consisted of low tidal volume ventilation (6–8 mL/kg), individualized positive end-expiratory pressure (PEEP) titration, maintaining ΔP below 15 cmH_2_O, and reducing MP.

### 2.3. Data Collection

Demographic variables, including age, sex, height, weight, body mass index (BMI), admission diagnosis, and comorbidities, along with the Acute Physiology and Chronic Health Evaluation (APACHE) II score (recorded 24 h post-admission), Sequential Organ Failure Assessment (SOFA) scores on the first day of ICU admission, duration of mechanical ventilation, and respiratory mechanics parameters (expiratory tidal volume [TV_e_], respiratory rate [RR], PEEP, peak pressure [P_peak_], compliance [C], driving pressure [ΔP], and mechanical power [MP]), were retrieved from electronic medical records. The Metavision Clinical Information System automatically records patient data from upstream devices, including mechanical ventilators, monitors, and infusion systems, and extracorporeal devices on a minute-by-minute basis. To standardize data acquisition, our team integrated specific formulas and algorithms into the Metavision system, enabling automatic calculation of key respiratory mechanics such as ΔP, MP. These parameters are continuously updated and stored in the database without requiring manual calculation. Minute-level data were extracted using structured query language (SQL) queries and subsequently aggregated into hourly and daily values for analysis. This automated approach ensured uniformity and accuracy in respiratory mechanics measurements across the entire study period. The hourly mechanical ventilation parameters in the Excel dataset were calculated at 24 h intervals (days) using the LEFT function of the Excel program as follows:

It was calculated by taking the difference in the first 10 characters of the signal date (Time 1) of the parameters transferred to the software from the mechanical ventilator and the first 10 characters of the patient’s admission to the ICU (Time 2) (=LEFT (Time 1;10) − (LEFT(Time 2;10)).

### 2.4. Calculation of Total Mechanical Power (MP_tot_)

For VCV mode, the volume control simplified equation (MPvcv−simpl) developed by Gattinoni et al. and for PCV, the pressure control simplified equation (MPpcv−simpl) developed by Becher et al. was used in this study [8,9].

MP_totvcv-simpl_ = (0.098 × RR × TV_e_ × (P_peak_ − ΔP/2))

MP_totpcv-simpl_ = (0.098 × RR × TV_e_ × (PEEP + ΔP_insp_)).

### 2.5. Statistical Analysis

In the study, the homogeneity of variables was assessed using the Shapiro–Wilk normality test. Due to the non-homogeneous distribution of patient data, the Mann–Whitney U test was employed to compare non-survival groups. Frequency distributions and percentages of categorical variables (e.g., gender, comorbidities) were analyzed using the chi-square test, with Fisher’s exact test applied when chi-square assumptions were not met. Statistical representations included median values, interquartile ranges (IQR), counts, and percentages.

For the parameters MP, ΔP, P_peak_, PEEP, TVe, C, and RR, receiver operating characteristic (ROC) curve analysis was conducted using the scikit-learn machine learning library in Python (Version 2.1.2, Amsterdam, The Netherlands), and the area under the curve (AUC) was calculated for the generated ROC curves. Logistic regression models were constructed using the statsmodels Python module, with bootstrapping performed to compute AUC values. The optimal threshold value, determined by the highest Youden index (distinguishing survival from non-survival), was selected to establish cutoff values.

Patient data were categorized into two groups based on the cutoff values of the analyzed parameters: one group with values greater than or equal to the cutoff (GE) and another with values below the cutoff (LESS). Survival analysis was performed using the lifelines package in Python. Kaplan–Meier estimation was applied to calculate survival functions for each group, with 95% confidence intervals (CI) computed. Analyses of ICU length of stay (in days) and ventilator-free days (VFDs) were conducted, considering the observation period and the event of patient survival or mortality. For each group, the median ICU length of stay (half-life), defined as the time point at which 50% of the patient population had deceased, was reported.

To investigate the impact of the studied parameters on survival time and VFDs, Cox proportional hazards regression models were applied to the grouped parameters. Hazard ratios (HR), associated *p*-values, and 95% CI were provided for each parameter. Statistical significance was evaluated at the *p* < 0.05 level.

## 3. Results

**Figure 2:**
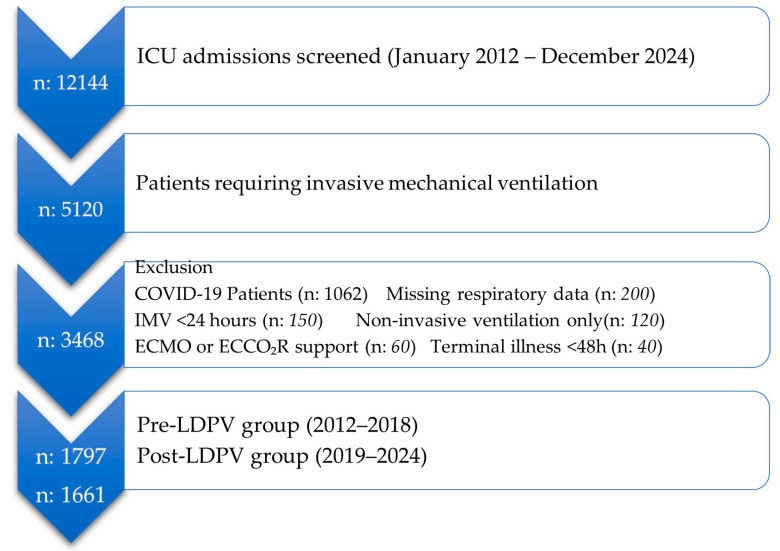
The study flowchart. ICU: Intensive care unit, IMV: Invasive mechanical ventilation, ECMO: Extracorporeal membrane oxygenation, ECCO_2_R: Extracorporeal carbon dioxide removal, LDPV: Lung and diaphragm protective ventilation.

A total of 5120 adult ICU patients received invasive mechanical ventilation during the 12-year study period. Following the application of exclusion criteria including ventilation for less than 24 h, missing data, non-invasive ventilation, extracorporeal support, terminal illness, pediatric age, and a COVID-19 diagnosis the final analytical cohort consisted of 3468 patients. The detailed selection process is illustrated in Figure 2. These patients were subsequently divided into a pre-LDPV implementation group (2012–2018, n = 1797) and a post-LDPV implementation group (2019–2024, n = 1661) for comparative analysis.

The demographic and baseline characteristics of the pre-LDPV and post-LDPV groups are presented in Table 1. The two groups were well-matched at baseline, exhibiting no statistically significant differences in age, sex, height, weight, BMI, or initial illness severity scores (SOFA and APACHE II). The post-LDPV group exhibited a statistically significant reduction in ICU mortality (41.1% vs. 47.7%, *p* < 0.0001), along with reductions in both the median length of ICU stay (8.8 vs. 11.0 days, *p* < 0.0001) and median ventilator-free days (6.0 vs. 7.2 days, *p* < 0.0001).

The distribution of admission diagnoses and comorbidities between the two cohorts is detailed in Table 2. There were no statistically significant differences in the primary reasons for ICU admission between the pre- and post-LDPV implementation periods. The most common admission diagnoses were pulmonary (26.2% vs. 27.6%), neurologic (26.3% vs. 25.5%), and gastrointestinal (25.4% vs. 23.7%). Similarly, the prevalence of key comorbidities, including diabetes mellitus, hypertension, malignancy, cerebrovascular disease, heart failure, coronary artery disease, liver failure, and chronic kidney disease, did not differ significantly between the 2012–2018 and 2019–2024 groups (all *p* > 0.05). The sole exception was a higher, though not statistically significant, proportion of patients with chronic obstructive pulmonary disease (COPD) in the pre-LDPV group (84.8% vs. 68.5%, *p* = 0.10).

Table 3 presents a comparative analysis of respiratory mechanics between the pre- and post-LDPV implementation periods. The adoption of the LDPV protocol was associated with statistically significant improvements across all measured ventilatory parameters. Specifically, the post-LDPV group (2019–2024) exhibited a significant reduction in d ΔP (12.9 [IQR 11.4–14.7] cmH_2_O vs. 14.3 [12.3–16.5] cmH_2_O; *p* < 0.0001) and MP (14.2 [12.1–16.4] J/min vs. 17.2 [14.1–21.3] J/min; *p* < 0.0001). Additionally, significant reductions were observed in TV_e_ (477 [434–520] mL vs. 511 [452–576] mL; *p* < 0.0001), P_peak_ (20.4 [17.9–23.1] cmH_2_O vs. 21.5 [18.6–24.5] cmH_2_O; *p* < 0.0001), and PEEP (6.8 [6.0–7.8] cmH_2_O vs. 7.0 [6.0–8.1] cmH_2_O; *p* = 0.0003). Concurrently, a significant improvement in respiratory system compliance was noted (39.9 [32.5–46.9] mL/cmH_2_O vs. 37.7 [30.0–46.3] mL/cmH_2_O; *p* < 0.0001), suggesting that the post-protocol cohort benefited from more protective and physiologically optimized ventilation strategies.

Table 4 provides a stratified comparison of respiratory mechanics between non-survivors and survivors within each study period. Across both the 2012–2018 and 2019–2024 groups, non-survivors consistently exhibited a statistically significant profile of more deleterious ventilatory parameters compared to survivors. This profile was characterized by significantly higher ΔP, MP, and P_peak_, alongside significantly lower C (all *p* < 0.0001). Notably, the association between elevated TV_e_ and mortality remained significant in both periods (*p* < 0.0001), whereas the relationship with RR was significant only in the pre-LDPV group (*p* < 0.0001). These findings persisted in the post-LDPV cohort, despite the adoption of more protective ventilation practices, indicating that increased mechanical stress remained a potent predictor of mortality irrespective of the study period.

Table 5, together with Figures S1 and S2 in the Supplementary Materials, demonstrates that protocol-driven reductions in key respiratory mechanics were closely associated with improved survival after LDPV implementation. In particular, lowering ΔP (median decrease of ~2 cmH_2_O in the post-LDPV group) significantly reduced mortality, with values ≥ 13.2 cmH_2_O still conferring a 37% higher hazard of death (HR 1.37, 95% CI 1.18–1.59). Similarly, reduced MP in the post-LDPV period was accompanied by better survival, although MP ≥ 15.3 J/min continued to predict excess mortality (HR 1.40, 95% CI 1.20–1.64), highlighting its dependence on ΔP as a major driver. Improvements in P_peak_ also translated into lower mortality, yet values above the post-LDPV cutoff (≥21.6 cmH_2_O) remained detrimental (HR 1.33, 95% CI 1.15–1.55). Compliance consistently emerged as a strong protective factor, with values below 34.0 mL/cmH_2_O associated with a 75% higher risk of death. In contrast, the prognostic effect of RR diminished after LDPV, reflecting that once ΔP and TV were optimized, tachypnea alone no longer conferred excess risk.

Table S1 in the Supplemental File extends the prognostic analysis to evaluate the probability of successful extubation, assessed through Cox regression for ventilator-free days. In the 2012–2018 group, elevated ΔP ≥ 15.2 cmH_2_O, HR 1.45, 95% CI 1.26–1.66), P_peak_ ≥ 23.5 cmH_2_O, HR 1.33, 95% CI 1.16–1.52), and RR ≥ 14/min, HR 2.06, 95% CI 1.79–2.36) were significantly associated with a reduced likelihood of extubation. Most notably, low C < 33.6 mL/cmH_2_O was a robust predictor of prolonged mechanical ventilation in both study periods (2012–2018: HR 2.06, 95% CI 1.53–2.77; 2019–2024: HR 1.97, 95% CI 1.73–2.25; *p* < 0.0001 for both). Following the implementation of LDPV, the hazards associated with ΔP, P_peak_ were attenuated and lost statistical significance.

## 4. Discussion

Following the implementation of the LDPV protocol at the end of 2018, a significant reduction in ICU mortality was observed in intubated patients, decreasing from 47.7% in the pre-implementation period (2012–2018) to 41.1% in the post-implementation period (2019–2024, *p* < 0.0001). This improvement persisted despite comparable baseline characteristics and slightly higher illness severity scores in the post-LDPV group, underscoring the clinical efficacy of systematic LDPV. In addition to reduced mortality, significant improvements were noted in ICU length of stay, further emphasizing the protocol’s positive impact on outcomes. Across both study periods, non-survivors consistently exhibited poorer respiratory mechanics, characterized by elevated ΔP, MP, TV_e_, P_peak_, alongside reduced C. Multivariable survival analyses confirmed that ΔP, MP, P_peak_, and TV_e_ above ROC derived thresholds were independent predictors of ICU mortality, while lower compliance was a strong predictor of both mortality and prolonged mechanical ventilation. Notably, protective changes in ventilatory parameters after 2019 suggest that protocolized monitoring and optimization of mechanical stress were key contributors to improved clinical outcomes. A 2022 retrospective ARDS cohort study reported ΔP > 14.5 cmH_2_O in non-obese patients as an independent mortality predictor (OR 1.04; *p* < 0.05) [10]. Similarly, in a large multicenter study HP et al. demonstrated that ΔP was independently associated with hospital mortality [11]. Our findings underscore the pivotal role of ΔP in determining clinical outcomes. Following the implementation of the LDPV protocol, median ΔP decreased by approximately 2 cmH_2_O (from 14.3 to 12.9 cmH_2_O), a change associated with a significant reduction in ICU mortality. This observation aligns with prior evidence, such as the work of Amato et al. which demonstrated that each unit increase in ΔP is independently associated with elevated mortality risk [6]. The correlation between improved survival and protocol-driven reductions in ΔP in our cohort reinforces the notion that systematic monitoring and optimization of ΔP are not only physiologically protective but also clinically significant in reducing mortality among mechanically ventilated patients. It is noteworthy that, despite the consistent prognostic value of ΔP, its measurement and clinical application at the bedside remain subjects of debate. Variability in calculation methods, the impact of spontaneous breathing activity, and uncertainties regarding its role as a direct therapeutic target have been highlighted in the recent literature [12]. These challenges underscore the need for additional prospective studies to optimize ΔP-guided strategies prior to their universal adoption in clinical practice.

Our analysis revealed a strong association between elevated MP and ICU mortality, with optimal cutoff values of ≥17.7 J/min in the pre-LDPV period. These thresholds align closely with findings from the multinational CHEST registry study, which reported that MP is associated with an adjusted odds ratio of 1.58 for ICU mortality [13]. Similarly, a Dutch analysis of non-ARDS patients confirmed MP as an independent predictor of 28-day mortality, even when accounting for other ventilatory parameters [14]. However, despite a reduction in median MP from 17 J/min in the pre-LDPV group to 15 J/min in the post-LDPV group, mortality remained elevated among patients exceeding the MP cutoff in both study periods. This suggests that ΔP, as the primary component of the MP formula, is the most critical determinant of mortality risk. The observed reduction in MP following LDPV implementation is largely attributable to a concurrent 2 cmH_2_O decrease in ΔP, underscoring ΔP as the key mechanistic driver of improved clinical outcomes.

Multiple studies have established that elevated inspiratory airway pressures, particularly P_peak_, are independently associated with increased mortality in mechanically ventilated patients. Sahetya et al. and Mamun et al. demonstrated that higher inspiratory pressures are associated with hospital mortality [15,16]. In our study, P_peak_ significantly decreased following the implementation of the LDPV protocol. However, patients with P_peak_ values exceeding the ROC-derived cutoff continued to exhibit higher mortality rates in both study periods. This finding underscores P_peak_ as a clinically significant marker of VILI and suggests that the observed reduction in P_peak_ contributed substantially to the improved survival outcomes following LDPV adoption.

Respiratory system C emerged as one of the predictors of mortality across both study periods. Patients with C values below the ROC-derived thresholds had significantly higher mortality, with cutoffs that remained nearly identical between the pre-LDPV (33.6 mL/cmH_2_O) and post-LDPV (34.0 mL/cmH_2_O) cohorts. Despite the overall improvements in ventilation practices after LDPV implementation, C-related mortality did not change substantially, indicating that C reflects underlying disease severity rather than modifiable ventilatory settings.

Our analysis demonstrated that PEEP ≥ 7 cmH_2_O was associated with increased ICU mortality in both the pre- and post-LDPV periods, although its statistical significance was diminished in the post-LDPV group. Given that the 10% of our patients included in the study had ARDS diagnosis, this finding highlights the importance of individualized PEEP settings rather than empiric application. Meta-analyses in non-ARDS ICU populations have similarly concluded that higher PEEP does not improve mortality or reduce ventilation duration, though it may decrease the incidence of ARDS and hypoxemia [17,18]. The LDPV protocol in our study emphasized PEEP titration, adjusted according to individual respiratory mechanics, which likely contributed to the attenuated association between PEEP and mortality observed in the post-LDPV period.

Our data highlight the prognostic importance of TV in ICU patients. Across both study periods, mortality was significantly elevated in patients ventilated with TV_e_ exceeding the ROC-derived thresholds, which generally surpass the protective range of approximately 6–8 mL/kg PBW. A comprehensive meta-analysis of ARDS trials published in 2022 demonstrated that low TV was associated with reduced 28-day mortality compared to higher TV (>8 mL/kg PBW), with a pooled risk ratio of 0.79 (95% CI 0.66–0.94), providing moderate evidence supporting LDPV strategies [19]. Furthermore, a large retrospective study of non-ARDS ICU patients reported that each 1 mL/kg PBW increase in tidal volume was associated with an 18% higher adjusted odds of mortality (OR 1.18, 95% CI 1.04–1.35; *p* = 0.010), underscoring the potential harm of even modest tidal volume elevations in non-ARDS populations [20]. Collectively, our study findings, consistent with these contemporary studies, emphasize that maintaining tidal volumes within physiologic, weight-based ranges is both feasible and critical for minimizing ventilator-associated mortality across diverse ICU populations.

In our study, RR > 14 breaths/min was significantly associated with increased ICU mortality during the pre-LDPV period, but this prognostic significance was lost in the post-LDPV group. This shift coincided with a protocol-driven reduction in ΔP, suggesting that the deleterious effects of tachypnea may be mediated through its contribution to VILI. By reducing ΔP, the LDPV strategy appears to have mitigated the mortality risk previously associated with elevated RR. This interpretation is supported by a recent scoping review by Aglen et al., which identified RR as one of the most sensitive indicators of patient deterioration and mortality in hospitalized adults [21]. Mechanistically, an elevated RR increases minute ventilation, potentially exacerbating dynamic hyperinflation, dead space ventilation, and mechanical power, thereby heightening the risk of lung injury when ΔP is not controlled. However, when ΔP and tidal volumes are maintained within protective ranges, as observed in the post-LDPV era, an elevated RR may primarily reflect compensatory responses to metabolic or gas exchange demands rather than directly contributing to mortality. These findings emphasize the importance of evaluating RR in the context of broader respiratory mechanics, particularly ΔP, rather than in isolation.

### Strengths and Limitations

This study possesses several notable strengths. It encompasses a large cohort spanning 12 years, with detailed, minute-level ventilatory data automatically captured through the electronic ICU system, facilitating precise calculations of ΔP, MP, and other respiratory mechanics. The study design enabled evaluation of the real-world impact of implementing a structured LDPV protocol across a heterogeneous ICU population. Additionally, the integration of ROC analysis and survival modeling provided clinically relevant cutoff values for key ventilatory parameters (ΔP, MP, P_peak_, TV_e_, C, and PEEP), enabling robust prognostic assessment. Notably, our cutoff thresholds closely aligned with values reported in large-scale international studies, reinforcing the external validity and generalizability of our findings.

Several limitations of this study warrant acknowledgment. First, the retrospective, single-center design restricts the generalizability of our findings, and causal relationships cannot be definitively established. Second, although the observed reduction in ΔP and associated improvements in clinical outcomes strongly suggest a causal relationship, the potential for residual confounding from unmeasured variables cannot be ruled out. Third, while C was a consistent predictor of mortality, it is influenced by underlying lung pathology and may not represent a directly modifiable therapeutic target. Similarly, the associations of PEEP and RR with mortality should be interpreted with caution, particularly given that the majority of patients in our cohort did not have ARDS. Fourth, the exclusion of COVID-19 patients, while necessary to maintain cohort homogeneity, limits the applicability of our findings to ARDS populations associated with pandemics. Finally, we acknowledge that temporal trends in respiratory parameters are inherently influenced by the introduction of the LDPV protocol, which may introduce methodological bias. However, this reflects real-world implementation, and the consistent prognostic impact of ΔP and MP across both periods strengthens the validity of our findings. Although the reduction in ΔP strongly correlated with improved survival, mortality in ICU patients is multifactorial, and residual confounding from non-ventilatory factors cannot be entirely excluded. Despite these limitations, the consistency of our results across multiple ventilatory parameters and their alignment with international evidence bolster the argument for prioritizing systematic ΔP-guided strategies in clinical practice.

## 5. Conclusions

This 12-year cohort study demonstrates that the implementation of a structured LDPV protocol achieved a reduction in ICU mortality, primarily through the systematic reduction in ΔP. MP and P_peak_, TV, RR, and PEEP provided additional prognostic insights. Collectively, these findings establish ΔP as the cornerstone of personalized LDPV strategies, with MP and related parameters serving as complementary markers to inform clinical decision-making.

## Figures and Tables

**Table 1:** Demographic and baseline characteristics of 2012–2018 and 2019–2024 groups.

	2012–2018 Group (n: 1797) Median (IQR_25–75_)	2019–2024 Group (n: 1661) Median (IQR_25–75_)	*p* Value
Age (year)	61 (44 to 75)	59 (49 to 69)	0.13 ^b^
Gender (female) (n) (%)	675 (37.5%)	663 (39.9%)	0.16 ^a^
Height (m)	170 (160 to 175)	170 (162 to 175)	0.55 ^b^
Weight (kg)	80 (70–85)	70 (80 to 86)	0.60 ^b^
BMI (kg/m^2^)	26.1 (24.2 to 29.4)	26.2 (24.2 to 29.2)	0.47 ^b^
SOFA	14.0 (10.0 to 15.0)	13.0 (11.0 to 14.0)	0.14 ^b^
APACHE II	18.0 (10.0 to 25.0)	19.0 (13.0 to 23.0)	0.83 ^b^
Ventilatory Free Days	7.2 (3.4 to 14.4)	6.0 (3.0 to 10.8)	<0.0001 ^b^*
ICU Length of Stay (day)	11.0 (6.1 to 20.7)	8.8 (5.5 to 14.4)	<0.0001 ^b^*
ICU Mortality (%)	858 (47.7%)	682 (41.1%)	<0.0001 ^b^*

*: Significant at 0.05 level, ^a^: Chi-square test, ^b^: Mann–Whitney U test. IQR: Interquartile range, BMI: Body mass index, SOFA: Sequential organ failure assessment score, APACHE: Acute physiology and chronic health evaluation score.

**Table 2:** Type of Diagnosis on Admission and Comorbidities of Patients in 2012–2018, 2019–2024 Groups.

Type of Admission n (%)	2012–2018 Group (n: 1797)	2019–2024 Group (n: 1661)	*p* Value
Pulmonary	467 (26.2%)	458 (27.6%)	0.29 ^a^
Neurologic	469 (26.3%)	423(25.5%)	0.67 ^a^
Gastrointestinal	453 (25.4%)	394 (23.7%)	0.31 ^a^
Cardiac	277 (15.5%)	219 (13.2%)	0.06 ^a^
Renal	72 (4.0%)	54 (3.3%)	0.27 ^b^
Metabolic	25 (1.4%)	19 (1.1%)	0.62 ^b^
Other	22 (1.2%)	35(2.1%)	0.0.06 ^b^
**Comorbidities n (%)**	**2012–2018 Group (n: 1797)**	**2019–2024 Group (n: 1661)**	***p* Value**
COPD	1513 (84.8%)	1134 (68.5%)	0.10 ^a^
Diabetes Mellitus	72 (4.0%)	84 (5.1%)	0.16 ^b^
Hypertension	72 (4.0%)	90 (5.4%)	0.06 ^b^
Malignancy	32 (1.8%)	43(2.6%)	0.13 ^b^
Cerebrovascular disease	36 (2.0%)	51 (3.1%)	0.06 ^b^
Heart failure	24 (1.3%)	37 (2.2%)	0.06 ^b^
Coronary artery disease	11 (0.6%)	21 (1.3%)	0.07 ^b^
Liver failure	13 (0.7%)	24 (1.4%)	0.058 ^b^
Chronic kidney disease	6 (0.3%)	15 (0.9%)	0.053 ^b^
Other	5 (0.3%)	13 (0.8%)	0.068 ^b^

^a^: Chi-square test, ^b^: Fisher’s exact test; COPD: Chronic obstructive pulmonary disease.

**Table 3:** Comparison of Respiratory Parameters of Patients in 2012–2018, 2019–2024 Groups.

Respiratory Mechanics	2012–2018 Group (n: 1797) Median (IQR_25–75_)	2019–2024 Group (n: 1661) Median (IQR_25–75_)	*p* Value
Respiratory rate (per minute)	14.0 (14.0 to 15.0)	14.0 (14.0 to 14.0)	<0.0001 *
PEEP (cmH_2_O)	7.0 (6.0 to 8.1)	6.8 (6.0 to 7.8)	0.0003 *
Expiratory Tidal Volume (mL)	511 (452 to 576)	477 (434 to 520)	<0.0001 *
P_peak_ (cmH_2_O)	21.5 (18.6 to 24.5)	20.4 (17.9 to 23.1)	<0.0001 *
Compliance (mL/cmH_2_O)	37.7 (30.0 to 46.3)	39.9 (32.5 to 46.9)	<0.0001 *
Driving Pressure (cmH_2_O)	14.3 (12.3 to 16.5)	12.9 (11.4 to 14.7)	<0.0001 *
Mechanical Power (J/min)	17.2 (14.1 to 21.3)	14.2 (12.1 to 16.4)	<0.0001 *

* significant at 0.05 level. PEEP: Positive end-expiratory pressure, P_peak_: Peak inspiratory pressure.

**Table 4:** Comparison of Respiratory Mechanics of Non-survived and Survived Patients in 2012–2018, 2019–2024 Groups.

2012–2018 Group (n = 1797)	Non-Survived (n = 858) Median (IQR_25–75_)	Survived (n = 939) Median (IQR_25–75_)	*p* Value
P_peak_ (cmH_2_O)	20.2 (17.9 to 23.0)	23.1 (20.3 to 26.2)	<0.0001 *
Expiratory Tidal Volume (mL)	521 (465 to 592)	499 (440 to 561)	<0.0001 *
PEEP (cmH_2_O)	6.8 (5.7 to 7.9)	7.4 (6.3 to 8.7)	<0.0001 *
Respiratory Rate (per minute)	14.0 (14.0 to 17.0)	14.0 (14.0 to 14.0)	<0.0001 *
Compliance (mL/cmH_2_O)	40.4 (33.4 to 48.8)	33.6 (27.2 to 42.9)	<0.0001 *
Driving Pressure (cmH_2_O)	13.3 (11.7 to 15.3)	15.5 (13.2 to 17.7)	<0.0001 *
Mechanical Power (J/min)	16.2 (13.1 to 19.8)	18.8 (15.3 to 23.3)	<0.0001 *
**2019–2024 Group (n = 1661)**	**Non-survived (n = 682) Median (IQR_25–75_)**	**Survived (n = 979) Median (IQR_25–75_)**	***p* Value**
P_peak_ (cmH_2_O)	19.7 (17.5 to 22.2)	21.6 (19.2 to 24.7)	<0.0001 *
Expiratory Tidal Volume (mL)	482 (442 to 526)	469 (424 to 509)	<0.0001 *
PEEP (cmH_2_O)	6.7 (5.8 to 7.5)	7.1 (6.2 to 8.0)	<0.0001 *
Respiratory Rate (per minute)	14.0 (14.0 to 14.0)	14.0 (14.0 to 14.0)	0.6066
Compliance (mL/cmH_2_O)	41.4 (34.7 to 48.5)	36.5 (27.8 to 43.7)	<0.0001 *
Driving Pressure (cmH_2_O)	12.5(11.0 to 14.2)	13.7 (12.0 to 15.9)	<0.0001 *
Mechanical Power (J/min)	13.8 (11.7 to 15.9)	14.9 (12.6 to 17.2)	<0.0001 *

* significant at 0.05 level. P_peak_: Peak inspiratory pressure, PEEP: Positive end-expiratory pressure.

**Table 5:** ICU mortality of patients with respiratory mechanics parameters above (≥) and below (<) the cutoff values in the 2012–2018 and 2019–2024 groups.

Fraction of Survival	2012–2018 Group (n = 1797)				2019–2024 Group (n = 1661)			
Covariate	Cutoff	HR	95% CI	Log Rank *p*	Cutoff	HR	95% CI	Log Rank *p*
Mechanical Power (J/min)	≥17.7	1.39	1.22–1.59	<0.0001 *	≥15.3	1.40	1.20–1.64	<0.0001 *
Driving Pressure (cmH_2_O)	≥15.2	1.66	1.45–1.90	<0.0001 *	≥13.2	1.37	1.18–1.59	<0.0001 *
P_peak_ (cmH_2_O)	≥23.5	1.56	1.36–1.78	<0.0001 *	≥21.6	1.33	1.15–1.55	0.0002 *
PEEP (cmH_2_O)	≥7.0	1.24	1.08–1.42	0.002 *	≥7.4	1.17	1.00–1.73	0.048 *
TV_e_ (mL)	≥469	1.27	1.11–1.46	0.0007 *	≥503	1.60	1.39–1.84	<0.0001 *
Compliance (mL/cmH_2_O)	<33.6	1.67	1.46–1.91	<0.0001 *	<34.0	1.75	1.54–2.00	<0.0001 *
Respiratory Rate	≥14/min	1.51	1.33–1.73	<0.0001 *	≥14/min	0.78	0.60–1.01	0.057

* significant at 0.05 level. HR: Hazard ratio, CI: Confidence interval, P_peak_: Peak inspiratory pressure, PEEP: Positive end-expiratory pressure, TV_e_: Expiratory tidal volume.

## Data Availability

Research data is available and can be shared upon request.

## Supplementary Materials

The following supporting information can be downloaded at: https://www.mdpi.com/article/10.3390/medicina61091668/s1, Figure S1: ROC graphics of 2012-2018 group; Figure S2: ROC graphics of 2019-2024 group; Table S1: The 2012–2018 and 2019–2024 groups were analyzed using Cox regression to assess the probability of extubation for patients with mechanical power (MPtot), driving pressure (ΔPinsp), peak inspiratory pressure (Ppeak), positive end-expiratory pressure (PEEP), tidal volume (TVe), dynamic compliance (Cdyn), and respiratory rate (RR) values above (≥) or below (<) their respective cutoff thresholds.

## Abbreviations

The following abbreviations are used in this manuscript:

APACHE II	Acute Physiology and Chronic Health Evaluation II
ARDS	Acute Respiratory Distress Syndrome
BMI	Body Mass Index
C	Respiratory System Compliance
CI	Confidence Interval
COPD	Chronic Obstructive Pulmonary Disease
ΔP	Driving Pressure
ECMO	Extracorporeal Membrane Oxygenation
ECCO_2_R	Extracorporeal Carbon Dioxide Removal
HR	Hazard Ratio
ICU	Intensive Care Unit
IQR	Interquartile Range
LDPV	Lung and Diaphragm Protective Ventilation
MP	Mechanical Power
MPtot	Total Mechanical Power
OR	Odds Ratio
PCV	Pressure-Controlled Ventilation
PBW	Predicted Body Weight
PEEP	Positive End-Expiratory Pressure
P_peak_	Peak Inspiratory Pressure
ROC	Receiver Operating Characteristic
RR	Respiratory Rate
SOFA	Sequential Organ Failure Assessment
TVe	Expiratory Tidal Volume
VCV	Volume-Controlled Ventilation
VFD	Ventilator-Free Days
VILI	Ventilator-Induced Lung Injury
