# Supplementary material for: Driving Down Mortality: A 12-Year Retrospective Cohort Analysis of Mechanical Power and Driving Pressure in Ventilated ICU Patients
Source: Medicina (Kaunas). 2025 Sep 14;61(9):1668. doi: 10.3390/medicina61091668 (PMC12471722; doi:10.3390/medicina61091668)
Supplement: Supplementary file 1 [file medicina-61-01668-s001.zip › medicina-3790143-supplementary.pdf]

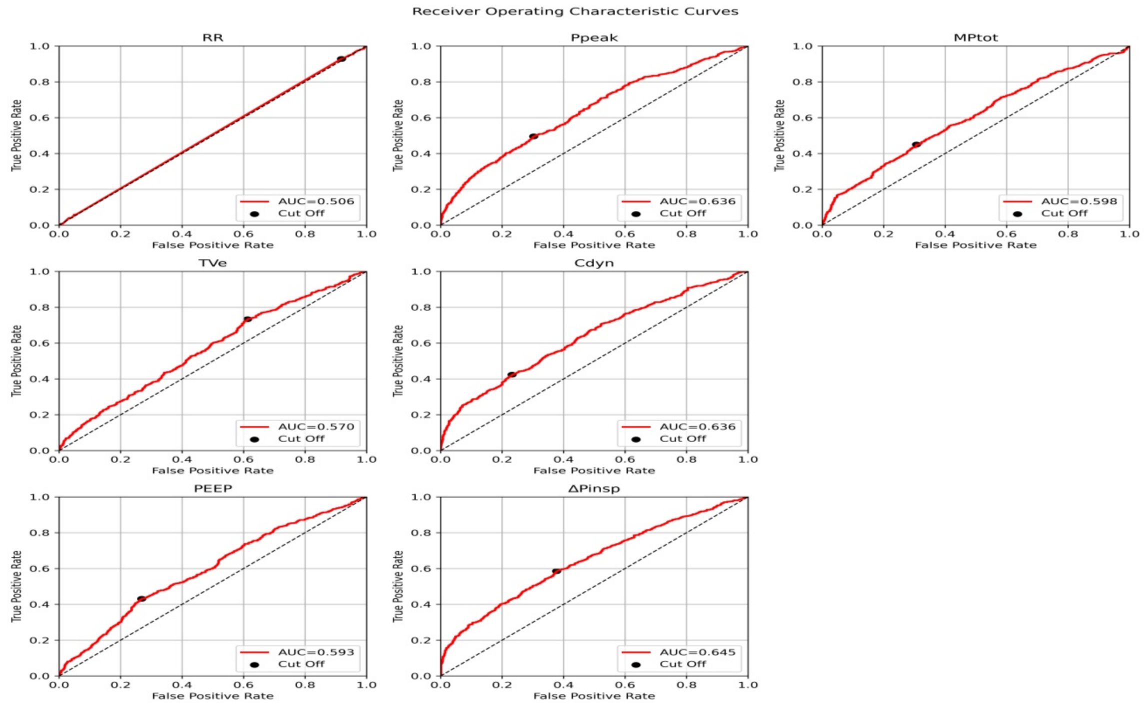

Figure S1. ROC graphics of 2012-2018 group. AUC: Area under curve, RR: Respiratory rate, P_{peak}: Peak inspiratory pressure, MP_{tot}: Total mechanical power, TV_e: Expiratory tidal volume, C_{dyn}: Dynamic complians, PEEP: Positive end-expiratory pressure, Δ_{insp}: Driving pressure.

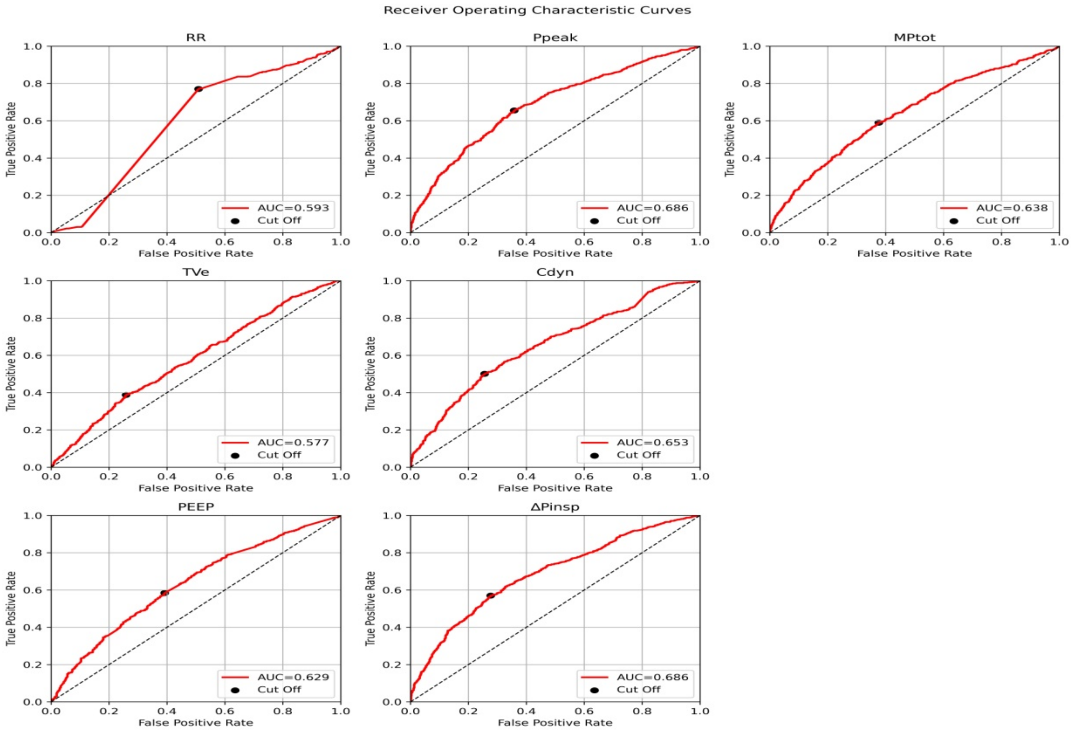

Figure S2. ROC graphics of 2019-2024 group. AUC: Area under curve, RR: Respiratory rate, P_{peak}: Peak inspiratory pressure, MP_{tot}: Total mechanical power, TV_e: Expiratory tidal volume, C_{dyn}: Dynamic complians, PEEP: Positive end-expiratory pressure, Δ_{insp}: Driving pressure.

Table S1. The 2012–2018 and 2019–2024 groups were analyzed using Cox regression to assess the probability of extubation for patients with mechanical power (MPtot), driving pressure (ΔPinsp), peak inspiratory pressure (Ppeak), positive end-expiratory pressure (PEEP), tidal volume (TVe), dynamic compliance (Cdyn), and respiratory rate (RR) values above (≥) or below (<) their respective cutoff thresholds.

**Probability of extubation**	**2012-2018 Group**				**2019-2024 Group**			
**VF days**	**Cut off**	**HR**	**95 % CI**	**Log rank P**	**Cut off**	**HR**	**95 % CI**	**Log rank P**
MP_{tot} (J/min)	≥ 17.7	1.17	1.02-1.34	0.024	**≥ 15.2**	1.15	0.99-1.34	0.068
ΔP_{insp} (cmH_2O)	≥ 15.2	1.45	1.26-1.66	<0.0001	**≥ 13.2**	1.10	0.94-1.28	0.233
P_{peak} (cmH_2O)	≥ 23.5	1.33	1.16-1.52	<0.0001	≥ 21.6	1.07	0.92-1.25	0.255
PEEP (cmH_2O)	<7.0	1.16	1.01-1.33	0.02	< 7.4	1.09	0.94-1.28	0.259
TV_e (ml)	≥469	1.27	1.11-1.47	0.0009	≥ 503	1.69	1.47-1.95	<0.0001
C_{dyn} (ml/cmH_2O)	<33.6	2.06	1,53-2,77	<0.0001	<34.0	1.97	1.73-2.25	<0.0001
RR	≥ 14/min	2.06	1.79-2.36	<0.0001	≥ 14/min	1.03	0.79-1.35	0.810

VF: Ventilatory free, HR: Hazard ratio, CI: Confidence Interval, Mptot: Total mechanical power, Δinsp: Driving pressure, Ppeak: Peak inspiratory pressure, PEEP: Positive end-expiratory pressure, TV_e: Expiratory tidal volume, Cdyn: Dynamic complians, RR: Respiratory rate,
